# Synthesis and Antiproliferative Effects of Amino-Modified Perillyl Alcohol Derivatives

**DOI:** 10.3390/molecules19056671

**Published:** 2014-05-22

**Authors:** Zi Hui, Meihui Zhang, Lin Cong, Mingyu Xia, Jinhua Dong

**Affiliations:** 1 Key Laboratory of Structure-Based Drug Design and Discovery, Ministry of Education, Shenyang Pharmaceutical University, Shenyang 110016, China; E-Mails: huizi781@163.com (Z.H.); zhangmeihui2005@gmail.com (M.Z.); 2 School of Life Science and Biopharmaceutics, Shenyang Pharmaceutical University, Shenyang 110016, China; E-Mail: conglin321@126.com

**Keywords:** perillyl alcohol, perillyl alcohol derivative, amino-modification, antiproliferation, apoptosis induction

## Abstract

Two series of amino-modified derivatives of (*S*)-perillyl alcohol were designed and synthesized using (*S*)-perillaldehyde as the starting material. These derivatives showed increased antiproliferative activity in human lung cancer A549 cells, human melanoma A375-S2 cells and human fibrosarcoma HT-1080 cells comparing with that of (*S*)-perillyl alcohol. Among these derivatives, compounds **VI_5_** and **VI_7_** were the most potent agents, with the IC_50_s below 100 μM. It was demonstrated that the antiproliferative effect of **VI_5_** was mediated through the induction of apoptosis in A549 cells.

## 1. Introduction

In recent decades natural products continue to attract intense attention due to their various bioactivities. Most of the drugs on the clinic market today are inspired by or derived from natural sources [1]. Perillyl alcohol, a naturally occurring monoterpene found in lavender, cherries and mint, has been suggested to be an effective agent against a variety of tumors as a farnesyl transferase (FTase) inhibitor [2,3,4,5,6,7]. Perillyl alcohol has been put into phase II clinical trials in cancer patients and the preliminary results indicate that this agent is well tolerated [8,9]. Since the potency of perillyl alcohol is modest compared to many antitumor agents [10], structural modification of perillyl alcohol has been carried out in recent years, and several kinds of perillyl alcohol derivatives have been synthesized. Among these derivatives, the perillyl alcohol carbamates, which were conjugated compounds of perillyl alcohol with some therapeutic agents, were found to be more active compounds [11], whereas other perillyl alcohol esters [12,13] and glucosides [14] were proved to be less potent than perillyl alcohol *in vitro*.

Amino-modification has been proved to be an efficient approach to increase water solubility and/or antitumor activity of several natural products, such as that of comptothecin [15,16], β-elemene [17] and limonene [18]. Thus, introduction of an amino moiety into the skeleton of perillyl alcohol might be favorable to improving antitumor activity. In this communication, two series of amino-modified derivatives of perillyl alcohol **IV**, **VI** were synthesized. Their activity of inhibiting tumor cell growth and the potential mechanism were studied in a few of cancer cell lines.

## 2. Results and Discussion

### 2.1. Synthesis of (S)-Perillyl Alcohol Derivatives

**Scheme 1:**
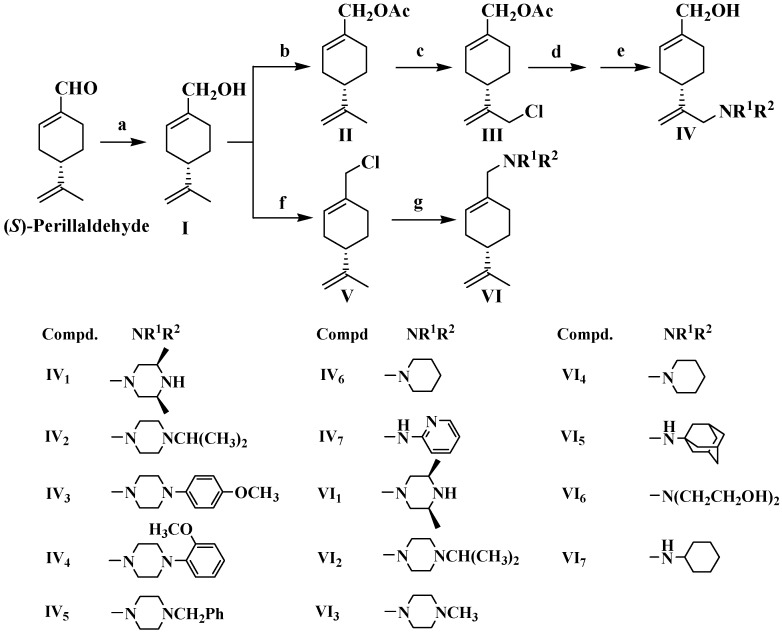
The synthetic routes and the substitutes of perillyl alcohol derivatives.

The synthetic route of the perillyl alcohol derivatives starting with (*S*)-perillaldehyde is outlined in Scheme 1, where the substituent groups are listed. (*S*)-Perillyl alcohol (**I**) was obtained from (*S*)-perillaldehyde via reduction with sodium borohydride.

*Reagents and conditions*: (a) NaBH_4_, EtOH, 0 °C-r.t., 3 h; (b) Ac_2_O, pyridine, r.t., 4 h; (c) NaClO, AcOH, 0 °C, 0.5 h; (d) R^1^R^2^NH, K_2_CO_3_, EtOH, reflux, 8–12 h; (e) NaOH, H_2_O, reflux, 2 h; (f) Ph_3_P, CCl_4_, CH_2_Cl_2_, r.t.; (g) R^1^R^2^NH, K_2_CO_3_, CH_3_CN, reflux, 6–8 h.

For a selective chlorination at the terminal allyl group of (*S*)-perillyl alcohol, acetylation of the hydroxyl group was carried out. The resulted perillyl acetate (**II**) was reacted with hypochloric acid, affording the intermediate **III**. The nucleophilic substitution of **III** with a heterocyclic amine or an aromatic amine and subsequent hydrolysis gave the target compound **IV**. The selective chlorination from (*S*)-perillyl alcohol (**I**) to the intermediate **V** was achieved in a mild condition via Appel reaction, avoiding the undesired chlorination of olefins by other reagents. The substitution reaction of **V** with an aliphatic amine or a heterocyclic amine gave the target compound **VI**.

### 2.2. Antiproliferative Effects in Tumor Cells

The cell growth inhibitory effect of these amino-modified derivatives was measured in A549, A375-S2 and HT-1080 cells using MTT assay. As shown in Table 1, the IC_50_s of (*S*)-perillyl alcohol in the three cells were more than 1,000 μM (the highest concentration used in this experiment). All the synthesized derivatives except **IV_6_** displayed much more potent cytotoxicity than (*S*)-perillyl alcohol. Among them, the two secondary aliphatic amines, **VI_5_** and **VI_7_**, were the most effective agents with the IC_50_s below 100 μM in the three tumor cells. Introduction of a substituted piperazinyl moiety (**IV_1_–IV_5_**, **VI_1_–VI_3_**) was of benefit to antiproliferative activity to some extent, but it did not show the same enhanced effect as that in the modification of β-elemene [17] and limonene [18]. It was found that the replacement of the hydroxyl group of (*S*)-perillyl alcohol with an amino moiety was more favorable to improving cytotoxic activity than the introduction of an amino moiety at the terminal allyl group, by comparing the IC_50_s of the two kinds of derivatives bearing the same substitutes (**VI_1_** *vs.* **IV_1_**, **VI_2_** *vs.* **IV_2_**, **VI_4_** *vs.* **IV_6_**).

**Table 1:** The antiproliferative effects of target compounds in A549, A375-S2 and HT1080 cells.

Compound	IC_50_ (μM)		
	A549	A375-S2	HT1080
**I**	>1000	>1000	>1000
**IV_1_**	437.76	309.61	556.38
**IV_2_**	948.35	501.32	>1000
**IV_3_**	405.07	287.79	59.91
**IV_4_**	403.22	110.07	386.35
**IV_5_**	384.63	395.10	340.43
**IV_6_**	735.29	>1000	>1000
**IV_7_**	417.03	436.77	73.11
**VI_1_**	270.11	359.23	426.01
**VI_2_**	427.52	463.80	409.16
**VI_3_**	393.69	472.75	483.37
**VI_4_**	619.11	90.05	761.80
**VI_5_**	53.80	53.80	56.17
**VI_6_**	432.46	424.74	496.41
**VI_7_**	69.50	72.77	69.37

The tumor cells were treated with a variety of concentrations of each compound for 48 h and the concentrations (IC_50_s) which inhibited 50% of cell growth were calculated. The data are presented as the mean of the results from three independent experiments.

To further verify the effect of compound **VI_5_**, we measured the viability of A549 cells treated with (*S*)-perillyl alcohol, amantadine, **VI_5_** or the combination of perillyl alcohol and amantadine (with the same concentrations) for 24 h. It was shown that the IC_50_ (152.72 μM) of **VI_5_** was 6-fold less than the IC_50_ (920.75 μM) of the combination of perillyl alcohol and amantadine while A549 cells treated with (*S*)-perillyl alcohol or amantadine alone didn’t reveal significant viability inhibition at concentration below 2,000 μM. The result indicated that the inhibitory effect of **VI_5_** was not caused by an additive effect or synergistic effect, but the specific structure of **VI_5_**.

### 2.3. Apoptosis in A549 Cells Induced by ***VI_5_***

**Figure 1:**
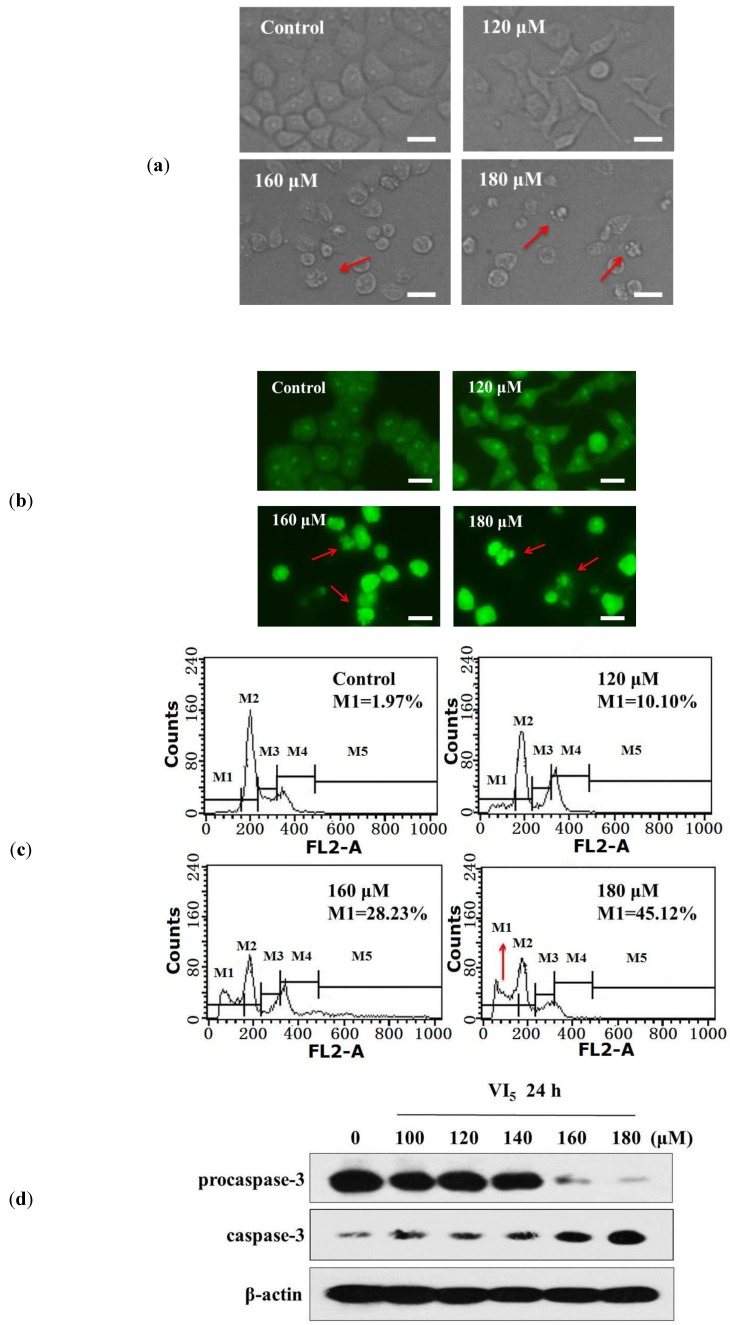
Apoptosis induced by **VI_5_** in A549 cells. (**a**) The cellular morphologic changes were examined using phase contrast microscopy; (**b**) The nuclear morphologic changes were observed using fluorescence microscopy after AO stained; (**c**) The cells stained with PI were analyzed by flow cytometric analysis; (**d**) The protein level of procaspase-3 was detected by western blot analysis.

As previously reported, perillyl alcohol could induce apoptosis in A549 cells [7]. Therefore, we examined whether the growth inhibitory effect of **VI_5_** was mediated through the induction of apoptosis. After treated with **VI_5_** for 24 h, significant morphologic changes were observed in A549 cells by phase contrast microscopy. Some of the cells showed membrane blebbing: a hallmark of apoptosis (Figure 1a). Meanwhile, compared with the control group, results of AO staining showed remarkable chromatin condensation and nuclear fragmentation in **VI_5_-**treated cells (Figure 1b). Flow cytometric analysis after PI staining revealed that the percentage of subG0/G1 ratio elevated from 1.97% to 45.12%, indicating that **VI_5_** induce apoptosis in a dose-dependent manner (Figure 1c). Caspase-3, a member of aspartate-specific cysteine proteases (caspase) family, has been considered as a key mediator of apoptosis [19]. Western blot results showed that the degradation of procaspase-3 increased after treatment with **VI_5_**, indicating that **VI_5_** could induce the activation of caspase-3 (Figure 1d).

## 3. Experimental

### 3.1. General Information

All reagents and solvents (analytical grade) were commercially available and used without further purification. Melting points were determined with a Yanaco micro melting point apparatus and were uncorrected. ^1^H-NMR spectra and ^1^^3^C-NMR spectrum were recorded in CDCl_3_ on a Bruker ARX-300 spectrometer. The coupling constants were recorded in hertz (Hz) and chemical shifts were reported in parts per million (δ, ppm) downfield from tetramethylsilane (TMS). High-resolution mass spectra (HRMS) were recorded on a high-resonance electrospray time-of-flight mass spectrometer LC/MSD QTOF 6520 (Agilent). Specific rotation was measured on a Perkin-Elmer 241 MC polarimeter (path length 1 cm). Column chromatography was performed on silica gel. Analytical TLC was performed on plates precoated with silica gel and iodine vapor was used to develop color on the plates.

### 3.2. (S)-(4-(Prop-1-en-2-yl)cyclohex-1-enyl)methanol (***I***)

To a solution of (*S*)-perillaldehyde (15.0 g, 0.1 mo1) in ethanol (100 mL) cooled to 0 °C, sodium borohydride (7.57 g, 0.2 mol) was added in portions. Then, the mixture was stirred at room temperature for 3 h. After the solvent was evaporated *in vacuo*, brine (30 mL) was added and the mixture was extracted with dichloromethane (3 × 30 mL). The combined organic extracts were washed with brine, dried over anhydrous sodium sulfate, and filtered. The filtrate was concentrated *in vacuo*. The residue was purified on a silica gel column with petroleum ether–ethyl acetate (3:1, R*_f_* = 0.51) as eluent to afford compound **I** as a colorless liquid (13.2 g, yield 86.8%). 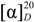: −86° (*c* = 1, MeOH), in lit. [20], 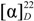: −88° (*c* = 1, MeOH). ^1^H-NMR δ: 5.70 (1H, br s, CH), 4.73, 4.71 (2H, s, s, CH_2_), 4.02–3.97 (2H, m, OCH_2_), 2.18–2.05 (4H, m), 2.00–1.93 (1H, m), 1.88–1.84 (1H, m), 1.74 (3H, s, CH_3_), 1.61–1.42 (1H, m). ^1^^3^C-NMR(75 MHz) δ: 149.8, 137.3, 122.5, 108.7, 67.3, 41.2, 30.4, 27.5, 26.1, 20.8 [21].

### 3.3. (S)-(4-(Prop-1-en-2-yl)cyclohex-1-enyl)methyl Acetate (***II***)

To a solution of compound **I** (12.1 g, 0.08 mol) in pyridine (25 mL), acetic anhydride (25 mL) was added dropwise. The mixture was stirred at room temperature for 4 h. The reaction was terminated by addition of methanol (2 mL), followed by addition of ethyl acetate (50 mL). The mixture was washed with aqueous sodium bicarbonate solution and brine. The organic layer was dried over anhydrous sodium sulfate, and filtered. The filtrate was concentrated *in vacuo*. The residue was purified on a silica gel column with petroleum ether–ethyl acetate (400:1, R*_f_* = 0.41) as eluent to afford compound **II** as a colorless liquid (14.8 g, yield 95.4%). ^1^H-NMR δ: 5.76 (1H, br s, CH), 4.73, 4.71 (2H,s, s, CH_2_), 4.46 (2H, s, OCH_2_), 2.24–1.80 (9H, m), 1.74 (3H, s, CH_3_), 1.52–1.44 (1H, m).

### 3.4. (S)-(4-(1-Chloroprop-2-en-2-yl)cyclohex-1-enyl)methyl Acetate (***III***)

To a mixture of compound **II** (11.6 g, 0.06 mol), acetic acid (5.4 g, 0.09 mol) and dichloromethane (150 mL) cooled to 0 °C, aqueous sodium hypochlorite solution (containing 10% available chlorine, 72 mL, 0.24 mol) was added dropwise. After the mixture was stirred for 0.5 h, saturated aqueous sodium sulfite solution (50 mL) was added and the mixture was extracted with dichloromethane (3 × 50 mL). The combined organic extracts were washed with brine, dried over anhydrous sodium sulfate, and filtered. The filtrate was concentrated *in vacuo*. The residue was purified on a silica gel column with petroleum ether–ethyl acetate (200:1, R*_f_* = 0.57) as eluent to afford compound **III** as a pale yellow liquid (11.78 g, yield 85.9%). ^1^H-NMR δ: 5.76 (1H, br s, CH), 5.19, 5.01 (2H, s, s, CH_2_), 4.46 (2H, s, OCH_2_), 4.10 (2H, s, CH_2_Cl), 2.45–2.42 (1H, m), 2.40–2.25 (1H, m), 2.20–1.80 (7H, m), 1.52–1.44 (1H, m).

### 3.5. (S)-1-Chloromethyl-4-(prop-1-en-2-yl)cyclohex-1-ene (***V***)

To a solution of compound **I** (9.0 g, 0.06 mo1) and triphenyl phosphine (31.5 g, 0.12 mol) in dichloromethane (50 mL) cooled to 0 °C, a mixed solution of carbon tetrachloride (12 mL) and dichloromethane (20 mL) was added dropwise. The mixture was stirred at room temperature overnight. Then, cyclohexane (200 mL) was added. The white solid precipitated was filtered. The filtrate was concentrated *in vacuo*. The residue was purified on a silica gel column with petroleum ether (R*_f_* = 0.68) as eluent to afford compound **V** as a pale yellow liquid (7.20 g, yield 70.4%). ^1^H-NMR δ: 5.83 (1H, br s, CH), 4.73, 4.71 (2H, s, s, CH_2_), 4.00 (2H, s, CH_2_Cl), 2.30–2.10 (4H, m), 2.05–1.84 (2H, m), 1.73 (3H, s, CH_3_), 1.61–1.42 (1H, m).

### 3.6. General Procedure for the Synthesis of Target Compounds ***IV_1_*–*IV_7_***

To a solution of compound **III** (0.9 g, 4 mmol) in ethanol (10 mL), potassium carbonate (1.10 g, 8 mmol) and amine (4.4 mmol) were added. The mixture was stirred and refluxed for 8–12 h. Then, aqueous sodium hydroxide solution (20%, 2 mL) was added and the resulting mixture was refluxed for another 2 h. The solvent was evaporated *in vacuo*. Brine (15 mL) was added to the residue and the mixture was extracted with dichloromethane (3 × 10 mL). The combined organic extracts were washed with brine, dried over anhydrous sodium sulfate, and filtered. The filtrate was concentrated *in vacuo*. The residue was purified on a silica gel column with dichloromethane–methanol (100:1 → 50:1 → 20:1) as eluent to afford the target product.

*(S)-(4-(3-(3,5-cis-Dimethylpiperazin-1-yl)prop-1-en-2-yl)cyclohex-1-enyl)methanol* (**IV_1_**): Yield: 62.7%; mp: 106-108 °C; R*_f_* = 0.30 (CH_2_Cl_2_/MeOH/Et_3_N: 200/10/1). ^1^H-NMR δ: 5.70 (1H, br s, CH), 4.95, 4.89 (2H, s, s, CH_2_), 4.00 (2H, s, OCH_2_), 3.05–2.85 (4H, m, NCH_2_, 2 × NCH), 2.81–2.70 (2H, m), 2.32–2.17 (2H, m), 2.15–1.83 (4H, m), 1.69–1.43 (3H, m), 1.12 (6H, d, *J* = 6.4, 2 × CH_3_); HRMS: *m*/*z* calcd. for C_16_H_29_N_2_O [M+H]^+^ 265.2280, found: 265.2274.

*(S)-(4-(3-(4-Isopropylpiperazin-1-yl)prop-1-en-2-yl)cyclohex-1-enyl)methanol* (**IV_2_**): Yield: 57.5%; mp: 52–53 °C; R*_f_* = 0.39 (CH_2_Cl_2_/MeOH/Et_3_N: 200/10/1). ^1^H-NMR δ: 5.71 (1H, br s, CH), 4.97, 4.89 (2H, s, s, CH_2_), 4.02 (2H, s, OCH_2_), 2.95 (2H, s, NCH_2_), 2.76–2.30 (9H, m, 4 × NCH_2_, NCH), 2.27–2.22 (1H, m), 2.17–2.09 (2H, m), 2.05–1.84 (3H, m), 1.57–1.50 (1H, m), 1.09 (6H, d, *J* = 6.5, CH(CH_3_)_2_); HRMS: *m*/*z* calcd. for C_17_H_31_N_2_O [M+H]^+^ 279.2436, found: 279.2430.

*(S)-(4-(3-(4-(4-Methoxyphenyl)piperazin-1-yl)prop-1-en-2-yl)cyclohex-1-enyl)methanol* (**IV_3_**): Yield: 54.6%; mp: 58–59 °C; R*_f_* = 0.49 (CH_2_Cl_2_/MeOH: 22/5). ^1^H-NMR δ: 6.93–6.79 (4H, m, Ar-H), 5.70 (1H, br s, CH), 5.00, 4.92 (2H, s, s, CH_2_), 4.00 (2H, s, OCH_2_), 3.76 (3H, s, OCH_3_), 3.18–2.93 (6H, m, 3 × NCH_2_), 2.65–2.45 (4H, m, 2 × NCH_2_), 2.37–1.86 (6H, m), 1.60–1.49 (1H, m); HRMS: *m*/*z* calcd. for C_21_H_31_N_2_O_2_ [M+H]^+^ 343.2385, found: 343.2381.

*(S)-(4-(3-(4-(2-Methoxyphenyl)piperazin-1-yl)prop-1-en-2-yl)cyclohex-1-enyl)methanol* (**IV_4_**): yield: 50.2%; pale yellow oil; R*_f_* = 0.47 (CH_2_Cl_2_/MeOH: 22/5). ^1^H-NMR δ: 7.04–6.79 (4H, m, Ar-H), 5.71 (1H, br s, CH), 5.01, 4.92 (2H, s, s, CH_2_), 4.01 (2H, s, OCH_2_), 3.86 (3H, s, OCH_3_), 3.19–2.89 (6H, m, 3 × NCH_2_), 2.79–2.40 (4H, m, 2 × NCH_2_), 2.38–1.84 (6H, m), 1.61–1.48 (1H, m); HRMS: *m*/*z* calcd. for C_21_H_31_N_2_O_2_ [M+H]^+^ 343.2385, found: 343.2380.

*(S)-(4-(3-(4-Benzylpiperazin-1-yl)prop-1-en-2-yl)cyclohex-1-enyl)methanol* (**IV_5_**): Yield: 61.3%; pale yellow oil; R*_f_* = 0.44 (CH_2_Cl_2_/MeOH: 22/5). ^1^H-NMR δ: 7.34–7.21 (5H, m, Ar-H), 5.69 (1H, br s, CH), 4.94, 4.88 (2H, s, s, CH_2_), 4.00 (2H, s, OCH_2_), 3.51 (2H, s, NCH_2_), 2.98–2.88 (2H, m, NCH_2_), 2.63–2.31 (8H, m, 4 × NCH_2_), 2.30–2.07 (4H, m), 2.01–1.83 (2H, m), 1.56–1.44 (1H, m); HRMS: *m*/*z* calcd. for C_21_H_31_N_2_O [M+H]^+^ 327.2436, found: 327.2431.

*(S)-(4-(3-(Piperidin-1-yl)prop-1-en-2-yl)cyclohex-1-enyl)methanol* (**IV_6_**): Yield: 51.3%; pale yellow oil; R*_f_* = 0.49 (CH_2_Cl_2_/MeOH/Et_3_N: 200/10/1). ^1^H-NMR δ: 5.70 (1H, br s, CH), 4.96, 4.87 (2H, s, s, CH_2_), 4.00 (2H, s, OCH_2_), 2.90 (2H, s, NCH_2_), 2.41–2.08 (8H, m), 2.01–1.84 (2H, m), 1.59–1.41 (7H, m); HRMS: *m*/*z* calcd. for C_15_H_26_NO [M+H]^+^ 236.2014, found: 236.2009.

*(S)-(4-(3-(Pyridin-2-ylamino)prop-1-en-2-yl)cyclohex-1-enyl)methanol* (**IV_7_**): Yield: 40.1%; pale yellow oil; R*_f_* = 0.39 (CH_2_Cl_2_/MeOH: 22/5). ^1^H-NMR δ: 8.07 (1H, d, *J* = 3.9, Ar-H), 7.46–7.39 (1H, m, Ar-H), 6.65–6.49 (1H, m, Ar-H), 6.36 (1H, d, *J* = 8.4, Ar-H), 5.71 (1H, br s, CH), 5.03, 4.92 (2H, s, s, CH_2_), 4.01 (2H, s, OCH_2_), 3.95–3.85 (2H, m, NCH_2_), 2.34–1.88 (6H, m), 1.62–1.51 (1H, m); HRMS: *m*/*z* calcd. for C_15_H_21_N_2_O [M+H]^+^ 245.1654, found: 245.1648.

### 3.7. General Procedure for the Synthesis of Target Compounds ***VI_1_*–*VI_7_***

To a solution of compound **V** (0.85 g; 5 mmo1) in acetonitrile (10 mL); potassium carbonate (1.04 g; 7.5 mmol) and amine (5.5 mmol) were added. The mixture was stirred and refluxed for 6–8 h. Then the solvent was evaporated *in vacuo*. Brine (15 mL) was added to the residue and the mixture was extracted with dichloromethane (3 × 10 mL). The combined organic extracts were washed with brine; dried over anhydrous sodium sulfate; and filtered. The filtrate was concentrated *in vacuo.* The residue was purified on a silica gel column with dichloromethane–methanol (100:1 → 50:1 → 20:1) as eluent to afford the target product **VI**.

*(S)-3,5-cis-Dimethyl-1-((4-(prop-1-en-2-yl)cyclohex-1-enyl)methyl)piperazine* (**VI_1_**): Yield: 54.1%; colorless oil; R*_f_* = 0.48 (CH_2_Cl_2_/MeOH/Et_3_N: 300/10/1). ^1^H-NMR δ: 5.59 (1H, br s, CH), 4.72, 4.71 (2H, s, s, CH_2_), 3.04–2.96 (2H, m, 2 × NCH), 2.81–2.73 (2H, m, NCH_2_), 2.38–2.13 (8H, m), 1.99–1.93 (1H, m), 1.85–1.79 (1H, m), 1.74 (3H, s, CH_3_), 1.49–1.42 (1H, m), 1.10 (6H, d, *J* = 6.0, 2 × CH_3_); HRMS: *m*/*z* calcd. for C_16_H_29_N_2_ [M+H]^+^ 249.2331, found: 249.2325.

*(S)-1-Isopropyl-4-((4-(prop-1-en-2-yl)cyclohex-1-enyl)methyl)piperazine* (**VI_2_**): Yield: 53.8%; pale yellow oil; R*_f_* = 0.55 (CH_2_Cl_2_/MeOH/Et_3_N: 300/10/1). ^1^H-NMR δ: 5.60 (1H, s, CH), 4.77–4.63 (2H, m, CH_2_), 2.86–2.76 (3H, m, NCH_2_, NCH), 2.69–2.42 (8H, m, 4 × NCH_2_), 2.15–1.91 (5H, m), 1.87–1.78 (1H, m), 1.73 (3H, s, CH_3_), 1.50–1.38 (1H, m), 1.10 (6H, d, *J* = 6.5, CH(CH_3_)_2_); HRMS: *m*/*z* calcd. for C_17_H_31_N_2_ [M+H]^+^ 263.2487, found: 263.2482.

*(S)-1-Methyl-4-((4-(prop-1-en-2-yl)cyclohex-1-enyl)methyl)piperazine* (**VI_3_**): Yield: 48.2%; pale yellow oil; R*_f_* = 0.50 (CH_2_Cl_2_/MeOH/Et_3_N: 300/10/1). ^1^H-NMR δ: 5.59 (1H, br s, CH), 4.70 (2H, s, CH_2_), 2.82(2H, s, NCH_2_), 2.54–2.29 (8H, m, 4 × NCH_2_), 2.28 (3H, s, NCH_3_), 2.25–1.79 (6H, m), 1.73 (3H, s, CH_3_), 1.54–1.33 (1H, m); HRMS: *m*/*z* calcd. for C_15_H_27_N_2_ [M+H]^+^ 235.2174, found: 235.2169.

*(S)-1-((4-(Prop-1-en-2-yl)cyclohex-1-enyl)methyl)piperidine* (**VI_4_**): Yield: 34.2%; pale yellow oil; R*_f_* = 0.44 (CH_2_Cl_2_/MeOH/Et_3_N: 300/10/1). ^1^H-NMR δ: 5.59 (1H, br s, CH), 4.72 (2H, s, CH_2_), 2.86–2.76(2H, m, NCH_2_), 2.38–2.23 (5H, m, 2 × NCH_2_, CH), 2.18–1.82 (5H, m), 1.75 (3H, s, CH_3_), 1.60–1.41 (7H, m) [22]; HRMS: *m*/*z* calcd. for C_15_H_26_N [M+H]^+^ 220.2065, found: 220.2060.

*(S)-N-((4-(Prop-1-en-2-yl)cyclohex-1-enyl)methyl)amantadine* (**VI_5_**): Yield: 36.2%; mp: 223–224 °C; R*_f_* = 0.32 (CH_2_Cl_2_/MeOH/Et_3_N: 300/10/1). ^1^H-NMR δ: 5.98 (1H, br s, CH), 4.71, 4.67 (2H, s, s, CH_2_), 3.49–3.40 (2H, m, NCH_2_), 2.29 (2H, m), 2.17–2.05 (11H, m), 1.96–1.82 (2H, m), 1.73–1.65 (9H, m), 1.51–1.45 (1H, m); HRMS: *m*/*z* calcd. for C_20_H_32_N [M+H]^+^ 286.2535, found: 286.2529.

*(S)-N-((4-(Prop-1-en-2-yl)cyclohex-1-enyl)methyl)diethanolamine* (**VI_6_**): Yield: 38.4%; colorless oil; R*_f_* = 0.34 (CH_2_Cl_2_/MeOH/Et_3_N: 300/10/1). ^1^H-NMR δ: 5.71–5.55 (1H, br, CH), 4.79–4.64 (2H, m, CH_2_), 3.63 (4H, t, *J* = 5.4, 2 × OCH_2_), 3.03 (2H, s, NCH_2_), 2.64 (4H, t, *J* = 5.4, 2 × NCH_2_), 2.17–1.81 (6H, m), 1.74 (3H, s, CH_3_), 1.53–1.39 (1H, m); HRMS: *m*/*z* calcd. for C_14_H_26_NO_2_ [M+H]^+^ 240.1963, found: 240.1957.

*(S)-N-((4-(Prop-1-en-2-yl)cyclohex-1-enyl)methyl)cyclohexanamine* (**VI_7_**): Yield: 35.4%; pale yellow oil; R*_f_* = 0.39 (CH_2_Cl_2_/MeOH/Et_3_N: 300/10/1). ^1^H-NMR δ: 5.63 (1H, br s, CH), 4.72, 4.70 (1H, s, s, CH_2_), 3.20 (2H, s, NCH_2_), 2.50–2.45 (1H, m, NCH), 2.15–2.06 (3H, m), 1.96–1.81 (4H, m), 1.73 (3H, s, CH_3_), 1.70–1.43 (4H, m), 1.28–1.13 (6H, m); HRMS;: *m*/*z* calcd. for C_16_H_28_N [M+H]^+^ 234.2222, found: 234.2216.

### 3.8. Biological Activity

*Cell culture*: Human lung cancer A549 cells, human melanoma A375-S2 Cells and human fibrosarcoma HT-1080 cells were obtained from American Type Culture Collection (ATCC, Manassas, VA, USA). A549 cells and HT1080 were cultured in DMEM medium with 10% fetal bovine serums (FBS, Tianjin Haoyang Biological Manufacture Co. LTD, Tianjin, China), 2 mM L-glutamine, 100 U/mL penicillin and 100 μg/mL streptomycin. A375-S2 cells were cultured in MEM medium with 10% fetal bovine serums, 2 mM L-glutamine, 100 U/mL penicillin and 100 μg/mL streptomycin at 37 °C in 5% CO_2_.

*MTT assay* [23]: Cells were planted in 96-well flat bottom micro titer plates (Corning, Tewksbury, MA, USA) with 7 × 10^3^ cells per well. After 24 h incubation, they were treated with the tested agents for the indicated times. After washing the plates with PBS, a 20 μL aliquot of 3-(4,5-dimethylthiazol-2-yl)-2,5-diphenyltetrazolium (MTT) solution (5.0 mg/mL) was added to each well and incubated for 3 h. The resulting crystal was dissolved in dimethyl sulfoxide. Optical density was detected by ELISA reader (Tecan, Salzburg, Austria). The percentage of cell viability inhibition was calculated as follows:

Inhibitory ratio (%) = (A_492_, control − A_492_, sample)/(A_492_, control − A_492_, blank) × 100% (1)

*Observation of morphologic changes*: A549 cells were treated with 0, 120, 160 and 180 μM **VI_5_** for 24 h on 24-well flat bottom plates. Then changes in cellular morphology were examined using phase contrast microscopy (Olympus, Tokyo, Japan).

*Acridine orange (AO) staining* [23]: A549 cells were treated with 0, 120, 160 and 180 μM **VI_5_** for 24 h on 24-well flat bottom plates. Then cells were washed with PBS, followed by incubation at room temperature with PBS containing 20 μg/mL AO for 15 min. The fluorescence of cells was observed using fluorescence microscopy.

*Flow cytometric analysis using propidium iodide (PI)* [24]: A549 cells were treated with 0, 120, 160 and 180 μM **VI_5_** for 24 h on 6-well flat bottom plates. The cells were harvested and washed by PBS and fixed by 70% cold ethanol at 4 °C for more than 18 h. After stained with 50 μg/mL PI and 1 mg/mL DNaseA-free RNaseA on ice in dark for 1 h, cells were analyzed on FACScan flow cytometer (Becton Dickinson, Franklin Lakes, NJ, USA).

*Western blot analysis* [24]: After treated with 0, 100, 120, 140, 160 and 180 μM **VI_5_** for 24 h, both adherent and floating cells were collected and lysed by Ultrasonic Cell Disruptor (Ningbo Scientz Biotechnology Co., Ltd, Ningbo, China) in whole cell lyse buffer (50 mM HEPES (pH 7.4), 1% Triton-X 100, 2 mM sodium orthovanadate, 100 mM sodiumfluoride, 1 mM edetic acid, 1 mM PMSF, 10 μg/mL aprotinin and 10 μg/mL leupeptin). The protein extracts was separated by 12% SDS-PAGE and transferred to PVDF membranes (Millipore, Billerica, MA, USA). After blocked with 5% skim milk, incubated with primary antibodies against procaspase-3 and caspase-3 at 4 °C overnight.

*Statistical assay*: All the presented data were confirmed at least three independent experiments. The data were analyzed by ANOVA using Statistics Package for Social Science SPSS software (version 13.0; SPSS, Chicago, IL, USA).

## 4. Conclusions

Two series of amino-modified derivatives of (*S*)-perillyl alcohol were designed and synthesized. The target compounds showed improved antiproliferative activity against A549, A375-S2 and HT-1080 cells. The structure-activity relationships revealed that the replacement of the hydroxyl group of (*S*)-perillyl alcohol with an amino moiety was more favorable to improving cytotoxic activity than the introduction of an amino moiety at the terminal allyl group. The antiproliferative effect of **VI_5_** was proved to be mediated through the induction of apoptosis in A549 cells.
